# Kallistatin inhibits tumour progression and platinum resistance in high-grade serous ovarian cancer

**DOI:** 10.1186/s13048-019-0601-6

**Published:** 2019-12-29

**Authors:** Huan Wu, Rongrong Li, Zhiwei Zhang, Huiyang Jiang, Hanlin Ma, Cunzhong Yuan, Chenggong Sun, Yingwei Li, Beihua Kong

**Affiliations:** 1 Department of Obstetrics and Gynecology, Qilu Hospital of Shandong University, 107 Wenhua Xi Road, Ji’nan, Shandong 250012 People’s Republic of China; 2 Shandong Key Laboratory of Gynecologic Oncology, Qilu Hospital of Shandong University, Ji’nan, Shandong People’s Republic of China

**Keywords:** Kallistatin, High-grade serous ovarian cancer, Proliferation, Metastasis, Platinum resistance, Apoptosis

## Abstract

Ovarian cancer is the most lethal gynaecologic malignancy. Although there are various subtypes of ovarian cancer, high-grade serous ovarian cancer (HGSOC) accounts for 70% of ovarian cancer deaths. Chemoresistance is the primary reason for the unfavourable prognosis of HGSOC. Kallistatin (KAL), also known as SERPINA4, is part of the serpin family. Kallistatin has been discovered to exert multiple effects on angiogenesis, inflammation and tumour progression. However, the roles and clinical significance of kallistatin in HGSOC remain unclear. Here, we showed that kallistatin was significantly downregulated in HGSOC compared to normal fallopian tube (FT) tissues. Low expression of kallistatin was associated with unfavourable prognosis and platinum resistance in HGSOC. Overexpression of kallistatin significantly inhibited proliferation and metastasis, and enhanced platinum sensitivity and apoptosis in ovarian cancer cells. Collectively, these findings demonstrate that kallistatin serves as a prognostic predictor and provide a potential therapeutic target for HGSOC.

## Introduction

Ovarian cancer is the most lethal gynaecologic malignancy and the fifth leading cause of female cancer deaths [1]. The 10-year survival is approximately 30% and has not improved significantly in the last decades [2]. Due to the late occurrence of symptoms, ovarian cancer is usually diagnosed at an advanced stage. In spite of high heterogeneity, high-grade serous ovarian cancer (HGSOC) deaths still account for three-quarters of total ovarian carcinoma deaths [3].Surgery and platinum-based chemotherapy remain the main treatments for HGSOC patients [4]. Recently targeted therapy has made significant progress in ovarian cancer, such as the vascular endothelial growth factor (VEGF) targeting drug bevacizumab and the p-oly-ADP-ribose polymerase (PARP) inhibitor olaparib [5–7]. Despite the high response to chemotherapy initially, the majority of advanced stage patients will relapse. Platinum resistance is one of the most challenging obstacles in prolonging the progression free interval (PFI) of HGSOC patients. The precise molecular mechanisms of HGSOC and platinum resistance are not fully understood.

Kallistatin (KAL), also known as SERPINA4, a member of the serpin family, was first identified as a tissue-kallikrein-binding protein in human serum in the 1980s [8, 9]. Subsequent studies revealed that kallistatin exerted multiple effects on angiogenesis, inflammation and tumour growth [10, 11]. Kallistatin is composed of two functional domains, the heparin-binding site and the active site [12]. Kallistatin inhibits VEGF-induced angiogenesis via the heparin-binding site [13]. The active site is essential for inhibiting tissue kallikrein’s activity [14]. Kallistatin has inhibitory effects in many malignancies such as hepatocellular carcinoma, gastric carcinoma and breast cancer [15–17]. However, the biological functions of kallistatin and its prognostic significance in ovarian cancer remain unclear.

In the present study, we aimed to illuminate the functions of kallistatin and the underlying mechanisms in ovarian cancer. We first evaluated the expression of kallistatin in HGSOC and normal fallopian tube (FT) tissues and analysed the association between expression and survival using a tissue microarray analysis. We then investigated the function of kallistatin in ovarian cancer cell proliferation, migration, invasion, platinum resistance and apoptosis.

## Materials and methods

### Tissue samples

A total of 312 HGSOC and 108 normal fallopian tube tissues were obtained in the Department of Obstetrics and Gynecology of Qilu Hospital, Shandong University, between 2003 and 2015. All the pathological results were confirmed blindly by two professional pathologists. Tumour stage was identified according to the International Federation of Gynecology and Obstetrics 2013 staging system [18]. A total of 108 normal fallopian tube (FT) tissues were collected from patients who underwent surgery with benign neoplasms at Qilu Hospital. The last date of follow-up was June 29, 2018. All patients within the study were informed and provided written consent. Platinum resistance was defined as tumour relapse or progression within 6 months. The study was approved by the Ethics Committee of Shandong University Qilu Hospital.

### Cell culture and reagents

OVCAR3 cells were purchased from American Type Culture Collection (ATCC). A2780 and A2780/DDP cells were gifts from Jianjun Wei’s laboratory. UWB1.289 and HEK293T cells were obtained from China Type Culture Collection. A2780, A2780/DDP and UWB1.289 cells were cultured in RPMI 1640 medium (Gibco, USA) with 10% foetal bovine serum (FBS) (Gibco, USA). OVCAR3 cells were cultured in RPMI 1640 medium with 20% FBS. HEK293T cells were cultured in DMEM (Gibco, USA) with 10% FBS. All cells were cultured at 37 °C under 5% CO_2_ in an incubator. Cisplatin was obtained from Sigma-Aldrich.

### Tissue microarray (TMA) construction and immunohistochemistry (IHC)

Sections of 4 μm were cut from each TMA receiver block, made by our laboratory. After deparaffinization in xylene and rehydration in a decreasing series of ethanol, slides were immersed in boiled 10 mmol/L EDTA buffer for antigen retrieval. Endogenous peroxidase was inactivated by 3% hydrogen peroxide for 15 min and nonspecific binding was blocked by goat serum for 30 min. Then the slides were covered with a kallistatin antibody (dilution 1:300, Abcam, USA, ab1544597) at 4 °C overnight, followed by incubation with an anti-rabbit antibody for 20 min. Finally, staining in the cytoplasm was evaluated by two pathologists who were blinded to the research. The four different scores used were defined as 0 (negative), 1 (weak), 2 (moderate), and 3 (strong), and the staining proportion ranged from 0 to 100 based on the percentage of stained cells. Kallistatin expression was graded by calculating the product-sum of the staining intensity and the proportion. The samples were divided into the low expression group if the product-sum was less than or equal to 110 and the high expression group if it was more than 110.

### Plasmid, lentivirus production, siRNA and transfection

The CDS sequence of kallistatin was purchased from Genechem (Shanghai, China) and inserted into the EcoRI/Nhel sites of the Plenti-C-Myc-DDK-IRES-Puro (PCMV) vector (Origene, USA). Lentivirus was produced by HEK293T cells with the psPAX2 (Addgene, USA) and pMD2.G (Addgene, USA) plasmids and Lipofectamine 2000 (Invitrogen, USA) according to the manufacturer’s protocol. After transfection with lentivirus for 24 h, the ovarian cancer cells were selected for a week in medium containing 4 μg/ml puromycin (Merck Millipore, USA) to obtain stable expressing cells. Small interfering RNA (siRNA) for silencing kallistatin was designed by Biosune (Shanghai, China) (sequence: 5′-CCAGCUUCGCGAUCAAAUUTT-3′). Ovarian cancer cells were transfected transiently with Lipofectamine 2000 (Invitrogen, USA).

### Protein extraction and western blot

The tissue samples and cells were placed on ice and treated with RIPA lysis buffer (Beyotime, China) containing NaF and PMSF. The concentration of proteins was quantified with a BCA Protein Assay kit (Merck Millipore, USA). A total of 60 μg of protein per well was separated with SDS-PAGE (5% stacking gel and 10–12% separation gel) and transferred to 0.22-μm PVDF membranes (Merck Millipore, USA) with the Bio-Rad Trans-blot system (16 V, 90 min). The membranes were blocked with 5% skim milk for 1 h and incubated with primary antibodies overnight at 4 °C. On the following day, the membranes were incubated with secondary antibodies for 1–2 h. The bands were detected with Western Lightening Plus-ECL reagent (GE, USA). GAPDH and ACTB were used as internal controls. ImageJ was used to analyze the bands.

### Cell proliferation assay

Cell proliferation was monitored by 4-[4,5-dimethythiazol-2-yl]-2,5-diphenyl tetrazolium bromide (MTT) assays. A total of 1000 ovarian cancer cells per well were seeded in 96-well plates in quintuplicate. Cell proliferation was measured at different times (1–6 days). For the assay, 20 μl of MTT (Sigma-Aldrich, USA) was added to each well at a designated time every day and incubated for 4 h at 37 °C. After careful removal of the supernatant, 100 μl of DMSO (Sigma-Aldrich, USA) was added per well. Then the absorbance values at 490 nm were measured by a microplate reader (ThermoScientific, USA). The experiment was performed in triplicate.

### Cell migration and invasion assay

For the assays, 1 × 10^5^–2 × 10^5^ cells were suspended in FBS-free medium and seeded into the upper chambers (8-μm pores, BD Biosciences, USA) of 24-well plates, and 700 μl of medium containing 20% FBS was added into the lower compartment. After an appropriate incubation time, we wiped away the cells adhered to the upper surface of the chambers. The cells adhered to the lower surface were fixed in methanol for 15 min and stained with 0.5% crystal violet for 15 min. The invasion assay was conducted in the same way except the filter membrane was covered with Matrigel (BD Biosciences, USA).

### Cell viability detection

A total of 3000 cells were seeded in 96-well plates in quintuplicate, and exposed to cisplatin at a series of concentrations (0, 2, 4, 8, 16, and 32 μg/ml) for 24 h after adhesion to plates. Then, 20 μl of MTT was added to each well and incubated for 4 h. The supernatant was exchanged with 100 μl of DMSO. The absorbance values at 490 nm were measured by a microplate reader. The experiment was performed in triplicate.

### Apoptosis

Ovarian cells were cultured in medium with cisplatin at a concentration of 2 μg/ml for 24 h. Then, the cells were trypsinized without EDTA, washed with 1 × phosphate buffer saline (PBS), centrifuged and resuspended in 1 × Annexin buffer and then stained with Annexin V-FITC and propidium iodide (PI) (BD Biosciences, USA). After 15 min of incubation, the cells were analysed with flow cytometry (BD Biosciences, USA). The experiment was performed in triplicate.

### In vivo nude mouse tumorigenesis

Four-week-old female BALB/c nude mice were purchased from NBRI of Nanjing University (Nanjing, China). UWB1.289 cells were transfected with PCMV-NC or PCMV-Kallistatin vector. To induce tumorigenesis, 5 × 10^6^ cells in 200 μl of 1 × PBS were injected subcutaneously into either side of the mouse axilla. After 3 weeks, the mice were sacrificed under anaesthesia and tumour weights were measured. All procedures performed in studies involving animals were in accordance with the National Institutes of Health guidelines for the care and use of Laboratory animals (NIH publication no. 8023, revised 1978). All animal experiments were approved by Shandong University Clinical Medical College Animal Experiment Ethics Committee.

### Statistical analysis

SPSS version 18.0 (Chicago, IL, USA) was used for the statistical analysis. Student’s t test was applied to assess the significance between two groups. The correlation between kallistatin expression and clinicopathologic parameters was analysed by the chi-squared test. Survival rates were calculated using the Kaplan-Meier method and the difference was calculated using log-rank test. Multivariate analysis of OS and PFS was performed by the Cox proportional hazard regression model. Additionally, *p* < 0.05, *p* < 0.01*,* and *p <* 0.001 were considered significant *, very significant ** and extremely significant ***, respectively.

## Results

### Expression of kallistatin was significantly downregulated in HGSOC

**Fig. 1:**
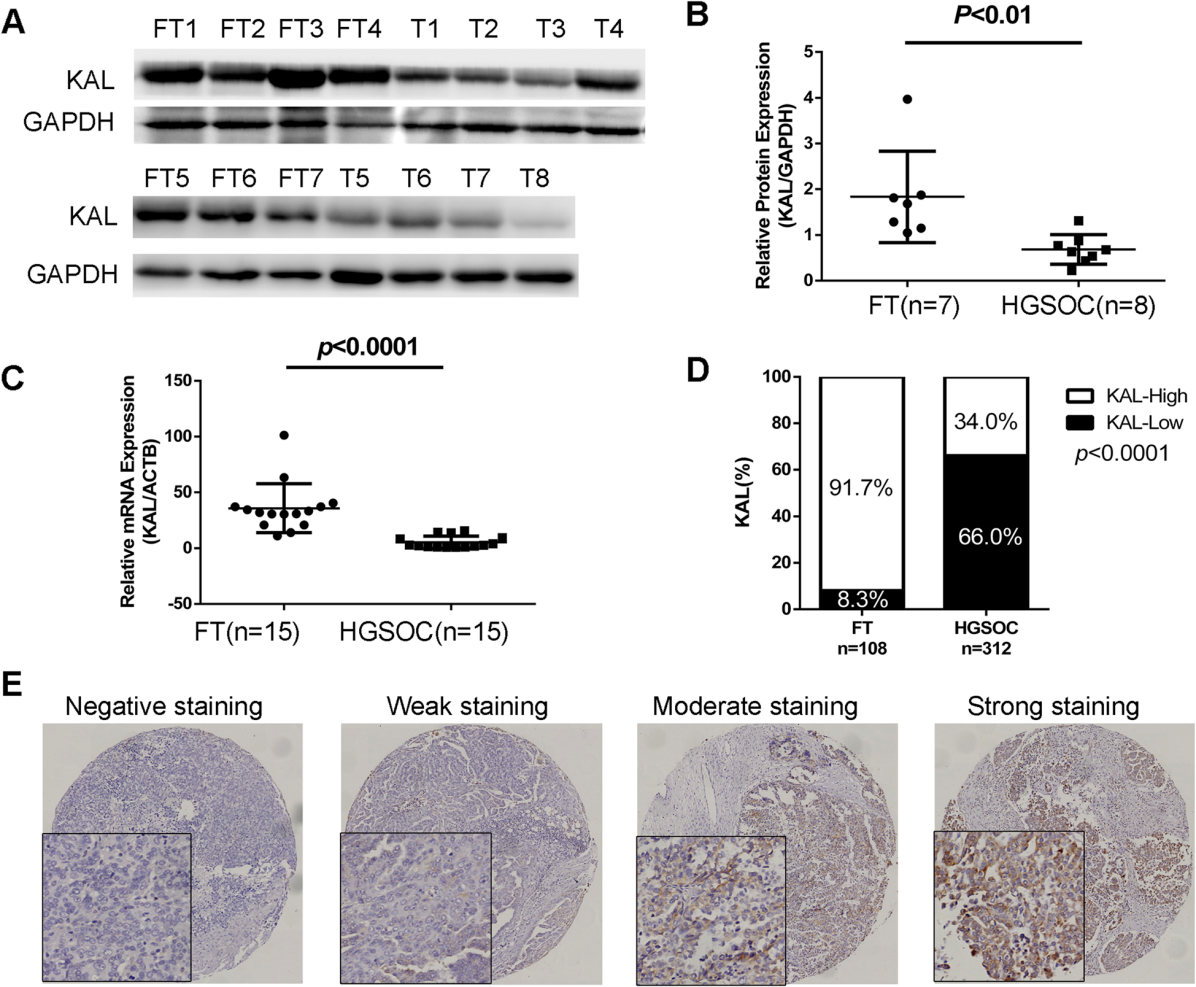
Kallistatin was downregulated in HGSOC tissues. (**a, b**) Kallistatin protein expression in normal fallopian tube (FT) tissues and HGSOC (T) tissues measured by western blot. (**c**) Kallistatin mRNA expression in 15 normal FT tissues and 15 HGSOC tissues measured by RT-PCR. (**d**) Kallistatin expression in normal FT tissues and HGSOC tissues measured by immunohistochemistry (IHC). (**e**) Representative IHC staining of kallistatin in the HGSOC TMA

We first analysed the protein level of kallistatin in human HGSOC (*n* = 8) and normal fallopian tube (FT, *n* = 7) tissues. The expression of kallistatin was significantly downregulated in HGSOC compared with FT tissues (*p* < 0.01, Fig. 1 a and b). We then identified the mRNA levels of kallistatin in 15 FT and 15 HGSOC tissues using RT-PCR (*p* < 0.0001, Fig. 1 c). To assess the expression pattern of kallistatin, we also performed IHC on TMAs (FT, *n* = 108, HGSOC, *n* = 312,). Higher expression of kallistatin was observed in FT (91.7%, 99/ 108 samples) than in HGSOC tissues (34%, 106/ 312 samples) (Fig. 1 d). Representative IHC staining of kallistatin in the HGSOC TMA is shown in Fig. 1 e.

### Low expression of kallistatin predicted an unfavourable prognosis of HGSOC

**Fig. 2:**
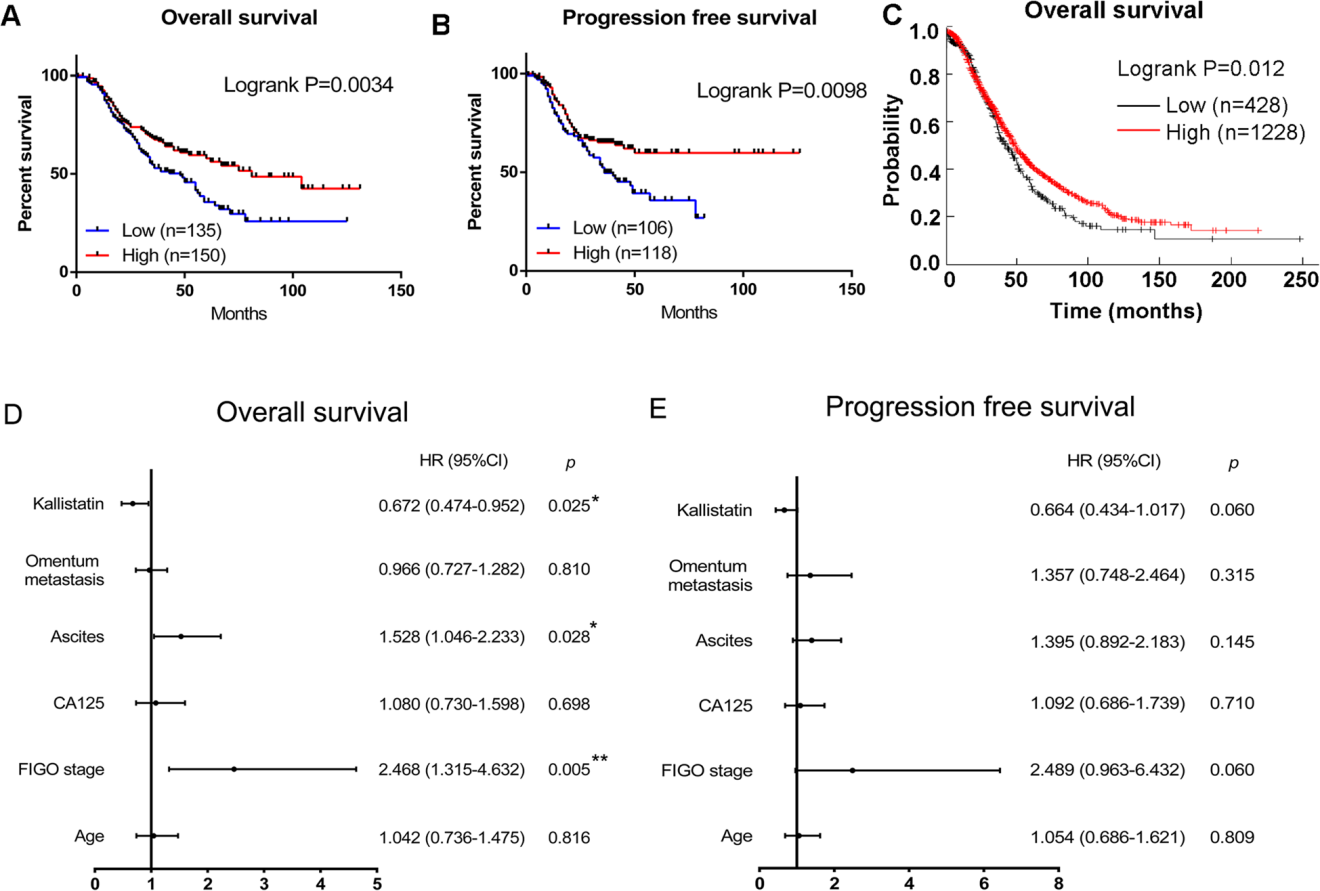
Low expression of kallistatin predicted poor prognosis of HGSOC. (**a**) Overall survival rates of HGSOC patients in the low versus high kallistatin expression group. (**b**) Progression free survival rates of HGSOC patients in the low versus high kallistatin expression group. (**c**) Overall survival analysis of serous ovarian cancer patients in data from the KM plotter database. (**d, e**) Forest plots depicting the results of the multivariate analysis of OS and PFS assessed by the Cox proportional hazard regression model. *p < 0.05, **p < 0.01, ***p < 0.001

According to our analysis, lower expression of kallistatin was associated with shorter OS (*p* = 0.0034) and PFS (*p* = 0.0098) (Fig. 2 a and b). In addition, we used the Kaplan-Meier-plotter [Ovarian Cancer] website to examine the association between overall survival and kallistatin expression in 1656 ovarian cancer patients [19]. Patients with higher kallistatin expression experienced longer overall survival times than patients with lower kallistatin expression (HR = 0.83, *p* = 0.012) (Fig. 2 c). In multivariate analysis of clinicopathologic features, the forest plot revealed that OS was significantly associated with kallistatin expression (HR = 0.672, 95% CI: 0.474–0.952, *p* = 0.025), ascites (HR = 1.528, 95% CI: 1.046–2.233, *p* = 0.028) and FIGO stage (HR = 2.468, 95% CI: 1.315–4.632, *p* = 0.005) (Fig. 2 d), while the results of the multivariate analysis of PFS were not significant (Fig. 2 e). Clinicopathologic parameter analysis revealed that kallistatin expression was correlated with age (*p* = 0.0170), volume of ascites (*p* = 0.0432), platinum resistance (*p* = 0.0127) and recurrence (*p* = 0.0156) (Table 1). The expression of kallistatin in platinum-resistant patients was significantly lower than that in platinum-sensitive patients, which suggested that kallistatin plays a role in the platinum resistance of HGSOC.

**Table 1:** Correlation between kallistatin expression and clinicopathologic parameters

Parameters		n	Kallistatin expression		*P* value
			Low (*n* = 144)	High (*n* = 168)	
Age(years)	< 55	149	58	91	0.0170*
	≥55	163	86	77	
FIGO stage	I + II	60	21	39	0.0614
	III + IV	250	122	128	
CA125 (U/ml)	< 600	122	57	65	0.6745
	≥600	174	77	97	
Ascites (ml)	< 1000	165	67	98	0.0432*
	≥1000	146	76	70	
Platinum	Resistant	91	30	15	0.0127*
	Sensitive	45	40	51	
Omentum metastasis	Positive	103	101	108	0.2081
	Negative	209	42	61	
Lymph node metastasis	Positive	72	29	27	0.2546
	Negative	56	30	42	
Recurrence	No	81	29	52	0.0156*
	Yes	181	94	87	

The clinicopathologic features of some patients were unable to be obtained, so some groups have fewer results than the total number of patients. However, the expression level of kallistatin in the omitted results was not different from the listed results. *p < 0.05, **p < 0.01, ***p < 0.001.

### Kallistatin inhibited the proliferation of ovarian cancer cells in vitro and in vivo

**Fig. 3:**
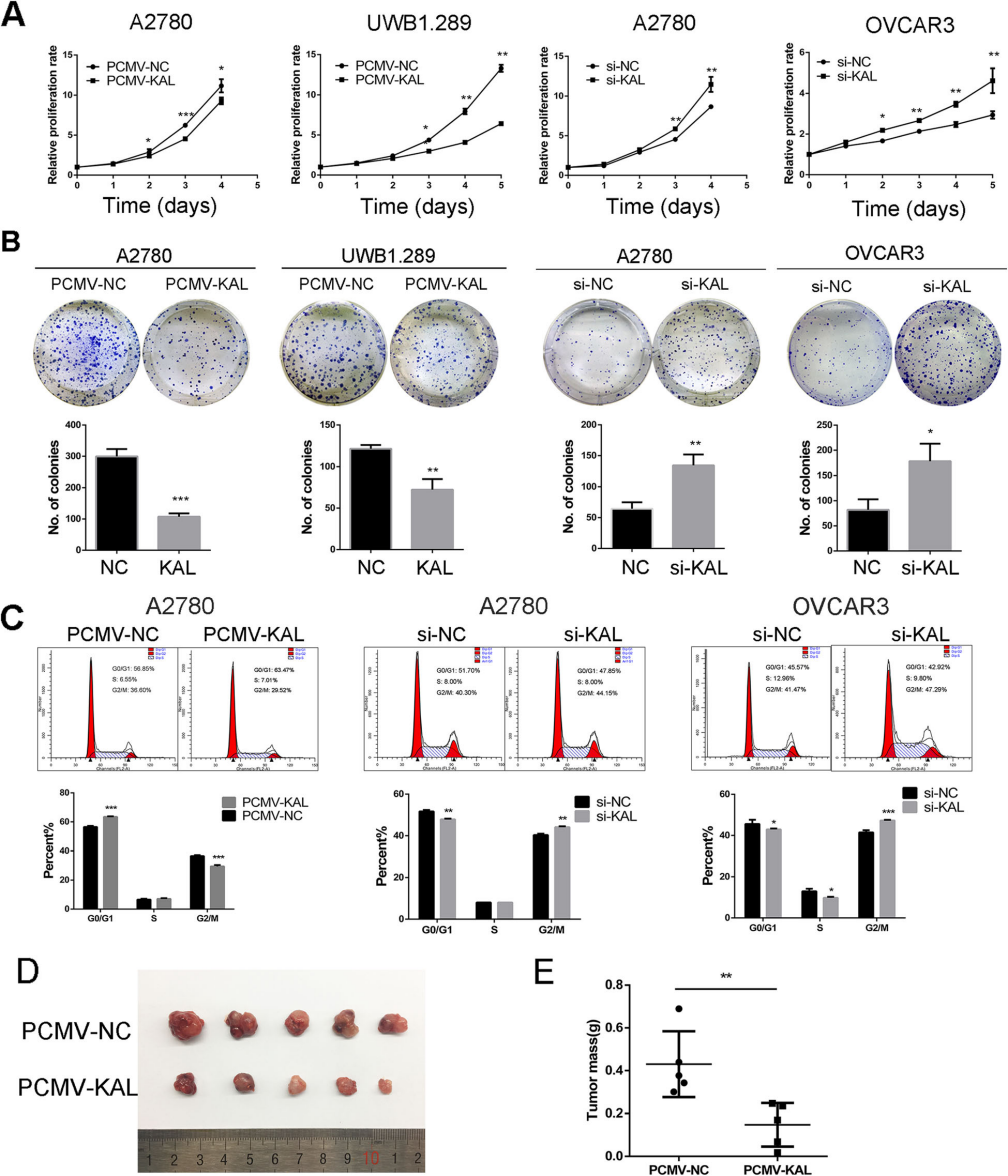
Kallistatin (KAL) inhibited the proliferation of ovarian cancer cells in vitro and in vivo. (**a**) The effect of kallistatin on ovarian cancer cell proliferation as measured by MTT assays; A2780 and UWB1.289 cells were transfected stably with PCMV-NC and PCMV-KAL. A2780 and OVCAR3 cells were transfected transiently with kallistatin siRNA. (**b**) Colony formation assays were used to measure the effect of kallistatin on A2780, UWB1.289 and OVCAR3 cell growth. (**c**) Cell cycle analysis of A2780 and OVCAR3 cells. (**d, e**) UWB1.289 cells stably transfected with PCMV-NC and PCMV-KAL were injected subcutaneously into nude female mice. The tumour weights in the PCMV-KAL group were significantly decreased compared with those in the control group. *p < 0.05, **p < 0.01, ***p < 0.001

To explore the biological significance of kallistatin in ovarian cancer, A2780 and UWB1.289 cells were transfected with PCMV-NC and PAMV-KAL to elevate the expression of kallistatin. OVCAR3 and A2780 cells were transfected transiently with kallistatin siRNA to decrease the expression. MTT assays and colony formation assays were performed and demonstrated that upregulation of kallistatin remarkably inhibited cell growth (*p* < 0.05, Fig. 3 a and b). Cell cycle analysis showed that overexpression of kallistatin increased the percentage of cells in the G1 phase and decreased the percentage of cells in the G2 phase, while kallistatin knockdown caused the opposite changes (Fig. 3 c). Based on findings in vitro, we subcutaneously injected UWB1.289 cells transfected with PCMV-NC and PCMV-KAL into nude mice. As shown in Fig. 3 d and e, overexpression of kallistatin significantly suppressed the tumorigenesis of ovarian cancer cells in vivo (0.430 ± 0.069 g vs. 0.148 ± 0.045 g, *p* = 0.009). IHC staining was utilized to detect kallistatin in the xenograft tissues. The expression of kallistatin was stronger in PCMV-KAL group than in the PCMV-NC group (Additional file 1: Fig. S1). These findings revealed that kallistatin exerted a growth-inhibiting function in ovarian cancer.

### Kallistatin inhibited the migration and invasion of ovarian cancer cells via inhibition of epithelial-mesenchymal transition (EMT)

**Fig. 4:**
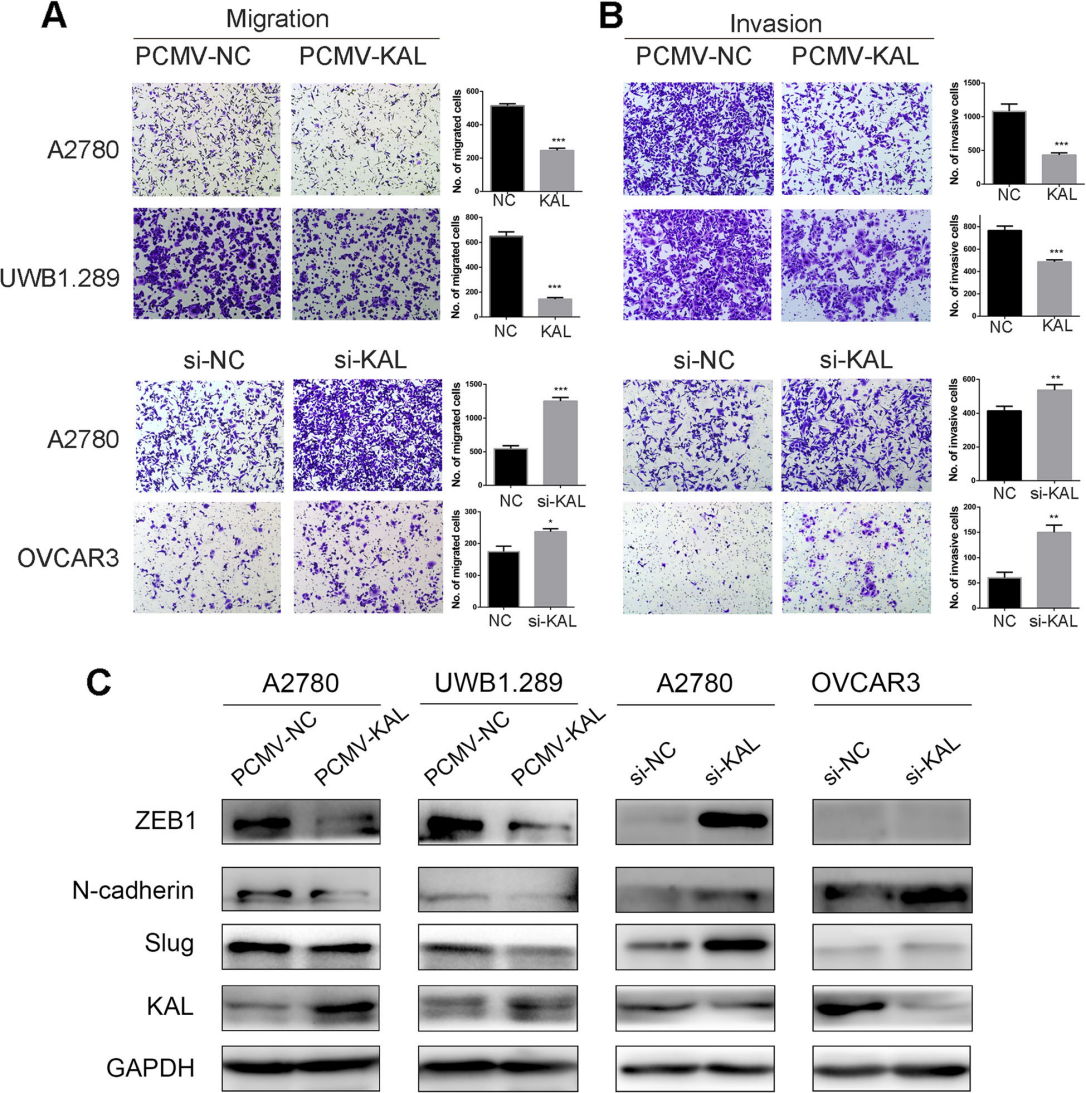
Kallistatin inhibited the migration and invasion of ovarian cancer cells in vitro. (**a, b**) Transwell assays were performed to measure the effect of kallistatin overexpression or knockdown on the migration and invasion of A2780, UWB1.289 and OVCAR3 cells. *p < 0.05, **p < 0.01, ***p < 0.001. (**c**) Western blot analysis of the EMT markers ZEB1, N-cadherin and Slug

The migration and invasion effects of kallistatin were analysed using transwell assays. As shown in Fig. 4 a and b, overexpression of kallistatin significantly impaired the migration and invasion abilities, while downregulation of kallistatin significantly promoted the migration and invasion abilities of A2780 and OVCAR3 cells. We further investigated the mechanism by analyzing EMT-related factors via western blot. The results revealed that overexpression of kallistatin downregulated N-cadherin, ZEB1 and Slug, which are mesenchymal biomarkers (Fig. 4 c). These data suggested that kallistatin suppressed cell metastasis by inhibiting EMT.

### Kallistatin enhanced sensitivity to cisplatin and apoptosis in ovarian cancer cells

**Fig. 5:**
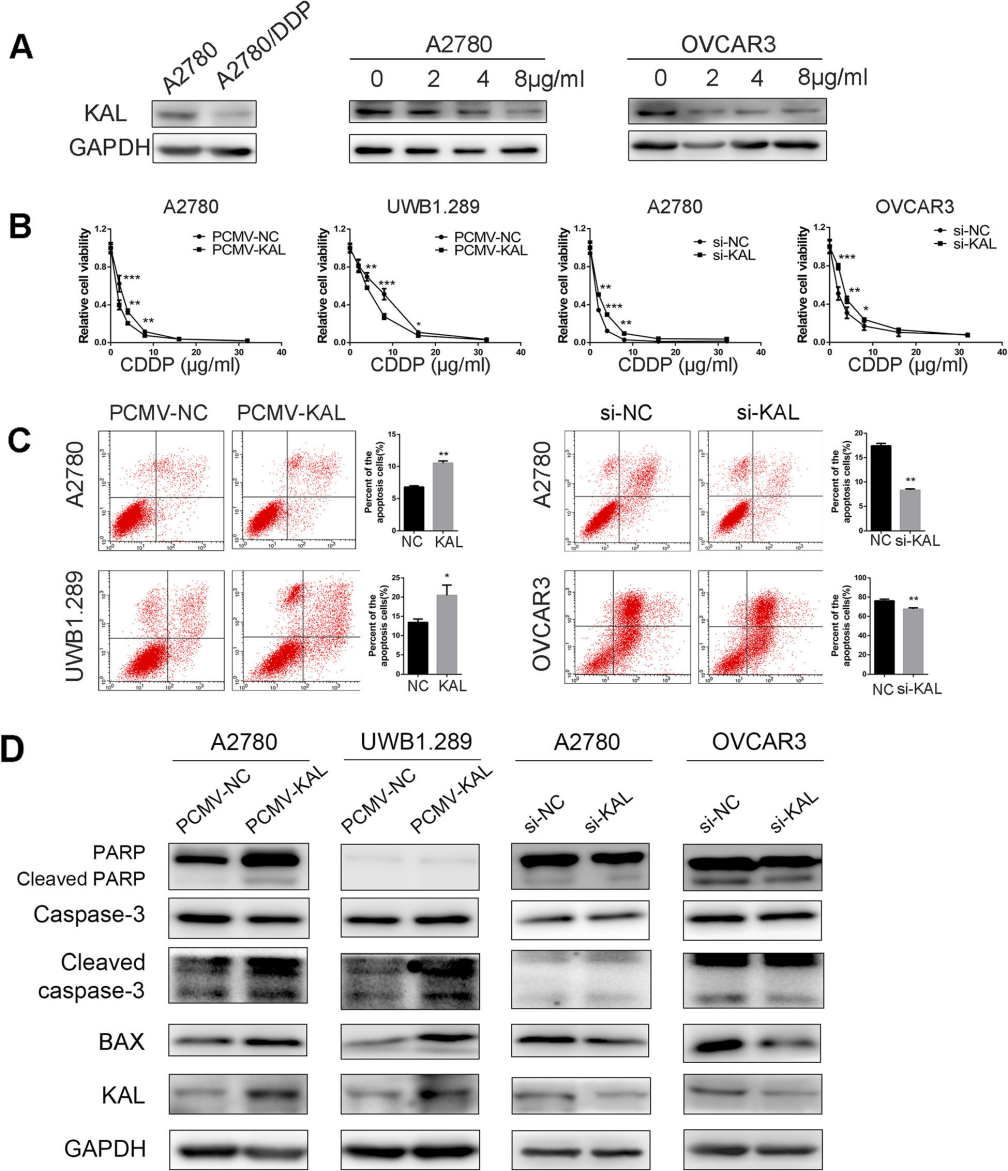
Kallistatin enhanced the platinum sensitivity of ovarian cancer cells. (A) Western blot analysis of kallistatin protein levels in A2780, A2780/DDP, A2780 and OVCAR3 cells treated with cisplatin at 0, 2, 4, and 8 μg/ml for 48 h. (B) Cell viability was determined using MTT in A2780, UWB1.289 and OVCAR3 cells. (C) The proportion of apoptotic cells was measured by Annexin V-FITC/PI staining and flow cytometry after cisplatin (CDDP) treatment for 24 h. (D) Western blot analysis of apoptosis-related proteins. *p < 0.05, **p < 0.01, ***p < 0.001

As shown in Table 1, low expression of kallistatin was associated with platinum resistance (*p* = 0.0127). The expression of kallistatin was decreased in cisplatin-resistant A2780/DDP cells compared to A2780 cells (Fig. 5 a). Correspondingly, there was a concentration-dependent decrease in kallistatin expression in A2780 and OVCAR3 cells that, which were exposed to cisplatin at 0, 2, 4, and 8 μg/ml for 48 h. The MTT assays revealed that cells with PCMV-KAL showed higher susceptibility to cisplatin than the control groups (Fig. 5 b). Clonogenic assays also confirmed that cells with kallistatin knockdown showed better ability to form colonies with the same dose of cisplatin than control cells (Additional file 1: Fig. S2). Apoptosis assays showed that overexpression of kallistatin significantly elevated the apoptotic cell fraction after 24 h of incubation with 2 μg/ml cisplatin (Fig. 5 c). To further investigate the role of kallistatin in apoptosis, we evaluated apoptosis-related proteins via western blot. As shown in Fig. 5 d, kallistatin stimulated the expression of cleaved PARP, cleaved Caspase-3 and Bax, which indicated that apoptosis was promoted. These findings further confirmed that kallistatin enhanced sensitivity to cisplatin.

## Discussion

Low expression of kallistatin has been confirmed in several malignancies [16, 17, 20, 21]. This study revealed for the first time that kallistatin was downregulated in ovarian cancer compared with fallopian tube tissues, and low expression of kallistatin was associated with unfavourable prognosis, platinum resistance and relapse in HGSOC. Previous studies have demonstrated that kallistatin is a reliable biomarker for liver cirrhosis and colorectal cancer [20, 22]. In our study, HGSOC patients with lower expression of kallistatin experienced shorter OS and PFS than HGSOC patients with higher expression of kallistatin, consistent with the KM-plotter database results. Multivariate analysis of clinicopathologic features indicated kallistatin can serve as a novel independent prognostic biomarker for HGSOC outcomes.

Kallistatin can suppress the proliferation of many malignant cells [16, 17, 23]. Consistent with these studies, our study found that upregulation of kallistatin caused an increase in cells in the G1 phase and a decrease in cells in the G2 phase and inhibited the growth of ovarian cancer cells in vitro and in vivo.

Lymph node metastasis and omentum metastasis contribute greatly to the relapse and death of patients with ovarian cancer. EMT has emerged as a critical regulator of metastasis in diverse malignancies, and it enhances mobility, invasion and resistance to apoptosis [24]. Furthermore, EMT has been identified to confer resistance to chemotherapy [25, 26]. Recent evidence revealed that kallistatin inhibited lymphatic metastasis in gastric cancer by downregulating VEGF-C expression [27]. Our data revealed that kallistatin overexpression can significantly suppress the metastasis and EMT of ovarian cancer cells, which might be one of the reasons for cisplatin resistance.

Resistance to platinum-based chemotherapy is one of the most challenging obstacles in prolonging PFI. It is estimated that over 80% of patients who respond initially to platinum will ultimately relapse at a certain stage [28]. As evidenced by our data, high kallistatin expression contributes to platinum sensitivity, indicating that the combination of platinum-based chemotherapy and kallistatin has the potential to lengthen PFI. Apoptosis, or programmed cell death, results in the orderly removal of damaged cells to maintain homeostasis and normal physical activities. Dysregulation of apoptosis contributes to not only tumour development but also tumour resistance to chemotherapy [29]. Kallistatin significantly reinforced cisplatin-induced apoptosis. Our study highlights the potential reversal of platinum resistance in ovarian cancer by kallistatin.

In summary, our findings indicate that kallistatin overexpression, which is associated with a favourable prognosis in HGSOC, can inhibit proliferation, metastasis, and chemotherapy resistance and enhance apoptosis. Kallistatin is a novel prognostic biomarker and a potential approach to increase chemotherapy efficacy in HGSOC.

## Supplementary information

**Additional file 1: Fig. S1.** HE staining and immunohistochemistry (IHC) staining of kallistatin in xenograft tumour tissues. **Fig. S2.** Colony formation assays were performed to evaluate the colony formation ability of A2780 and UWB1.289 cells treated with different doses of cisplatin. #*p* > 0.05, *p < 0.05, **p < 0.01, ***p < 0.001.

## Data Availability

The datasets generated during the current study are available from the corresponding author on reasonable request.
