# Correction to: Kallistatin inhibits tumour progression and platinum resistance in high-grade serous ovarian cancer

**DOI:** 10.1186/s13048-020-00628-5

**Published:** 2020-03-13

**Authors:** Huan Wu, Rongrong Li, Zhiwei Zhang, Huiyang Jiang, Hanlin Ma, Cunzhong Yuan, Chenggong Sun, Yingwei Li, Beihua Kong

**Affiliations:** 1 Department of Obstetrics and Gynecology, Qilu Hospital of Shandong University, 107 Wenhua Xi Road, Ji’nan, Shandong 250012 People’s Republic of China; 2 Shandong Key Laboratory of Gynecologic Oncology, Qilu Hospital of Shandong University, Ji’nan, Shandong People’s Republic of China

**Correction to: J Ovarian Res (2019) 12:125**

**https://doi.org/10.1186/s13048-019-0601-6**

The original article [1] contains errors in Fig. 3c, Results and Discussion. The picture of cell cycle analysis of A2780 si-KAL in the original publication was incorrectly a repeat of A2780 si-NC, and the correct picture of A2780 si-KAL is updated.

**Fig. 3:**
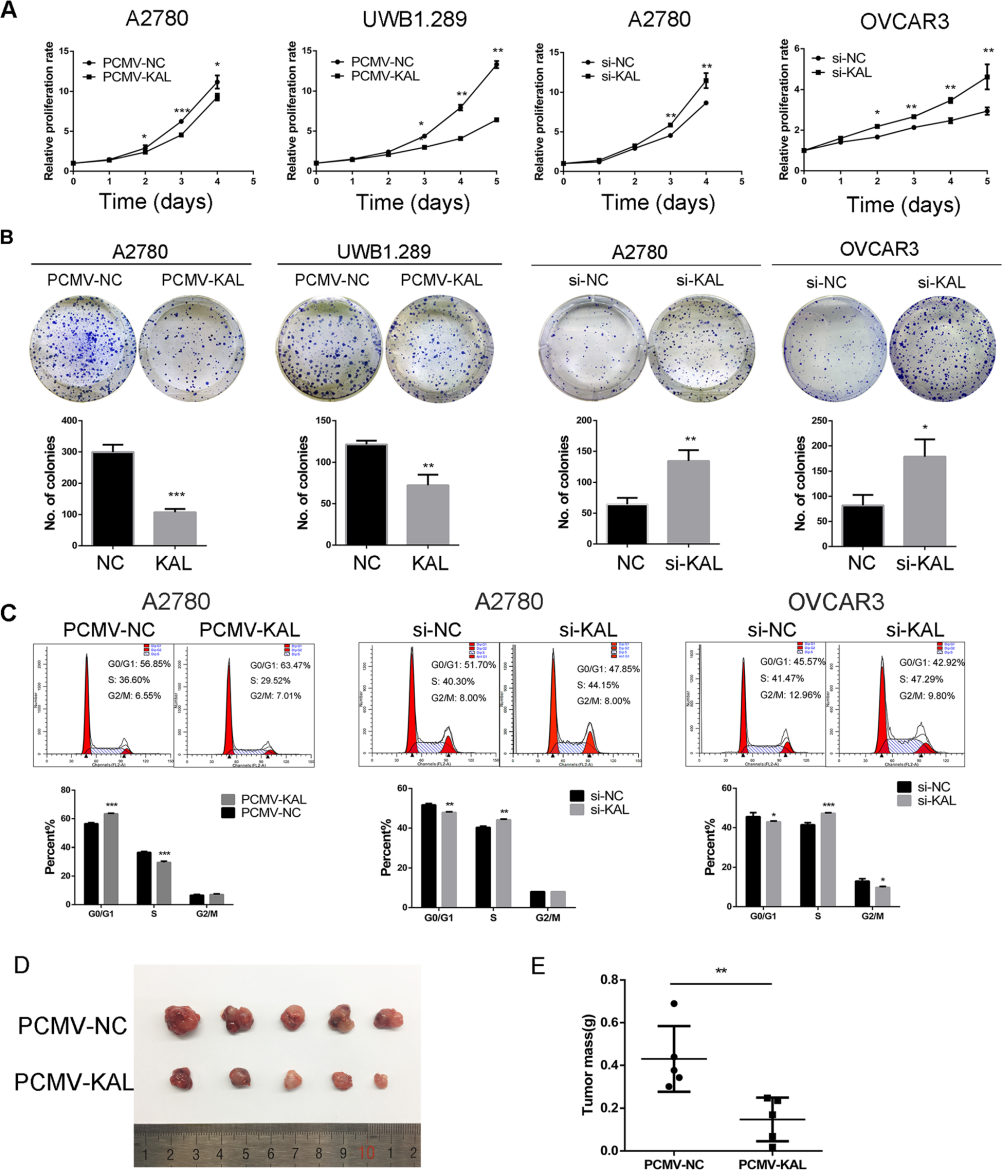
Kallistatin (KAL) inhibited the proliferation of ovarian cancer cells in vitro and in vivo. (**a**) The effect of kallistatin on ovarian cancer cell proliferation as measured by MTT assays; A2780 and UWB1.289 cells were transfected stably with PCMV-NC and PCMV-KAL. A2780 and OVCAR3 cells were transfected transiently with kallistatin siRNA. (**b**) Colony formation assays were used to measure the effect of kallistatin on A2780, UWB1.289 and OVCAR3 cell growth. (**c**) Cell cycle analysis of A2780 and OVCAR3 cells. (**d, e**) UWB1.289 cells stably transfected with PCMV-NC and PCMV-KAL were injected subcutaneously into nude female mice. The tumour weights in the PCMV-KAL group were significantly decreased compared with those in the control group. *p < 0.05, **p < 0.01, ***p < 0.001

Additionally, the data of G2/M phase and S phase are switched in Fig. 3c. Correspondingly, “G2 phase” in Results and Discussion of the original article (page 5, left column, line 16 and page 6, right column, line 22) should be changed to “S phase”, which still indicated that kallistatin inhibited the proliferation of ovarian cancer cells. The corrected Fig. 3 appears below.

The authors sincerely apologize for the errors. The errors do not affect the conclusion of the article.
