# Supplementary material for: Kallistatin inhibits tumour progression and platinum resistance in high-grade serous ovarian cancer
Source: J Ovarian Res. 2019 Dec 29;12:125. doi: 10.1186/s13048-019-0601-6 (PMC6935502; doi:10.1186/s13048-019-0601-6)

**Kallistatin inhibits tumour progression and platinum resistance in high-grade serous ovarian cancer**

Huan Wu ^a, b^, Rongrong Li ^a, b^, Zhiwei Zhang ^a, b^, Huiyang Jiang ^a, b^, Hanlin Ma ^a, b^, Cunzhong Yuan ^a, b^, Chenggong Sun ^a, b^, Yingwei Li ^a, b^, and Beihua Kong ^a, b*^

Huan Wu and Rongrong Li contributed equally to the work.

^a^ Department of Obstetrics and Gynecology, Qilu Hospital, Shandong University, Ji’nan, Shandong, P.R. China;

^b^ Gynecology Oncology Key Laboratory, Qilu Hospital, Shandong University, Ji’nan, Shandong, P.R. China

* Correspondence to: [kongbeihua@sdu.edu.cn](mailto:kongbeihua@sdu.edu.cn)

**Figure S1.** Hematoxylin-eosin (HE) staining and immunohistochemistry (IHC) staining of kallistatin in xenograft tumour tissues.

**Figure S2.** Colony formation assays were performed to evaluate the colony formation ability of A2780 and UWB1.289 cells treated with different doses of cisplatin. #*p* > 0.05, **p* < 0.05, ***p* < 0.01, ****p* < 0.001.

**Figure S1**


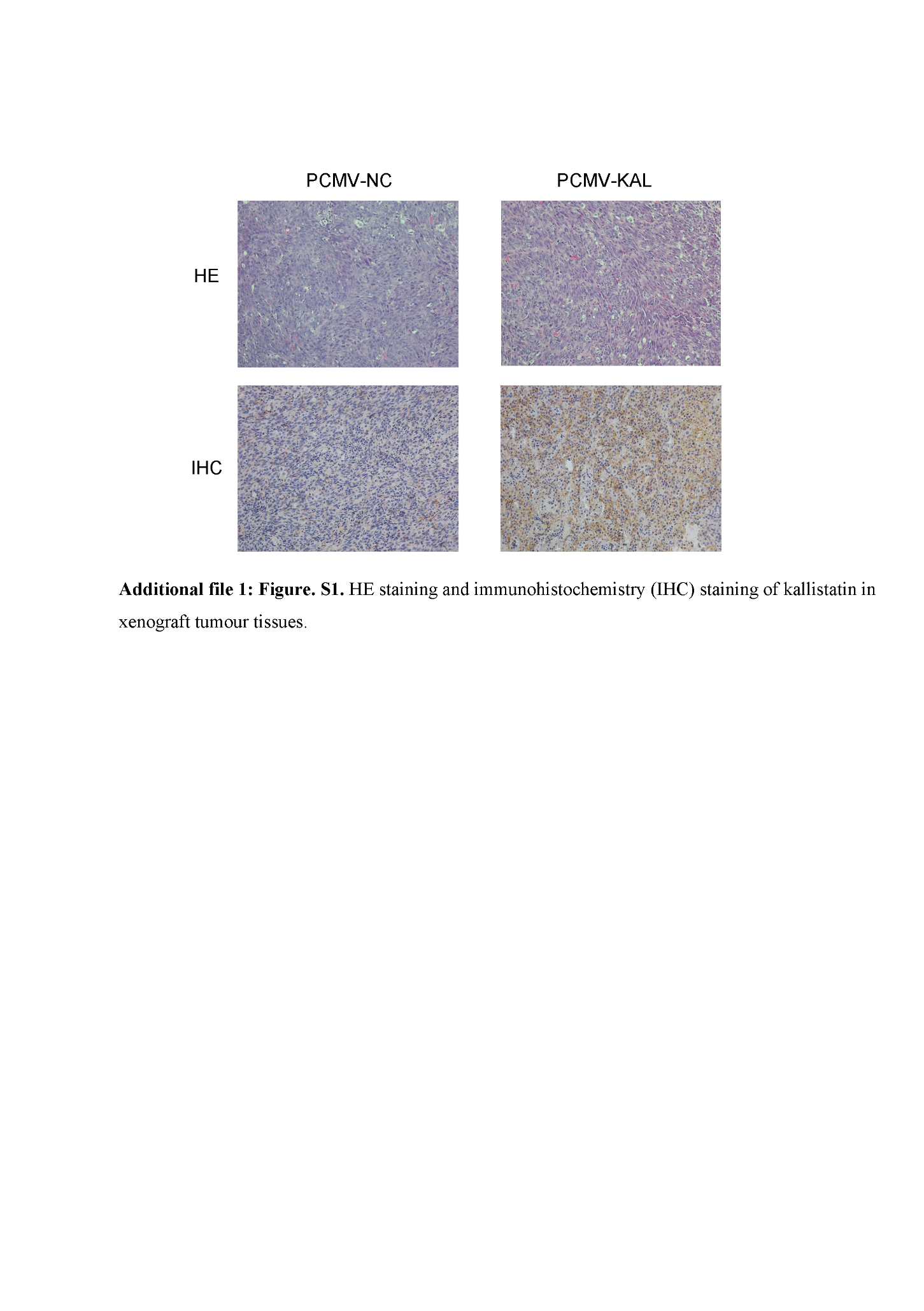


**Figure S2**


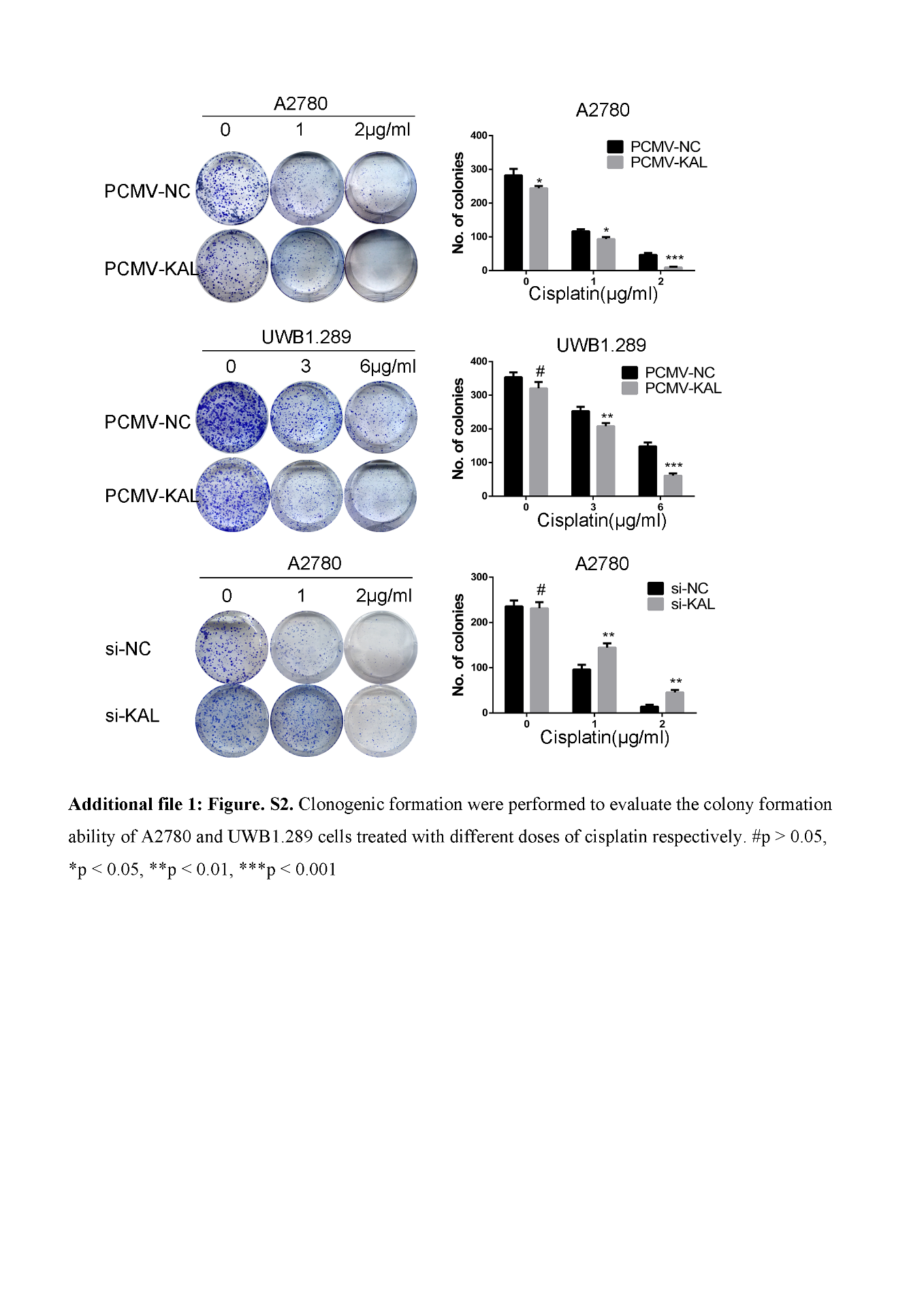

Supplement: Supplementary file 1 — Additional file 1: Fig. S1. HE staining and immunohistochemistry (IHC) staining of kallistatin in xenograft tumour tissues. Fig. S2. Colony formation assays were performed to evaluate the colony formation ability of A2780 and UWB1.289 cells treated with different doses of cisplatin. #p > 0.05, *p < 0.05, **p < 0.01, ***p < 0.001. [file 13048_2019_601_MOESM1_ESM.docx]
